# A hidden danger in cancer patients: intestinal parasites

**DOI:** 10.55730/1300-0144.6370

**Published:** 2026-06-12

**Authors:** Aslı GEÇGEL, Ayşe Merve PARMAKSIZOĞLU AYDIN, Ahmet TÜRK, Esra ERDEM KIVRAK, Gökhan VATANSEVER, Nevin TURGAY, Erdem GÖKER, Meltem TAŞBAKAN

**Affiliations:** 1 Division of Medical Oncology, Department of Internal Medicine, Faculty of Medicine, Ege University, İzmir, Turkiye; 2 Department of Infectious Diseases and Clinical Microbiology, Faculty of Medicine, Ege University, İzmir, Turkiye; 3 Department of Internal Medicine, Faculty of Medicine, Ege University, İzmir, Turkiye; 4 Department of Infectious Diseases and Clinical Microbiology, Faculty of Medicine, Celal Bayar University, Manisa, Turkiye; 5 Division of Parasitology, Department of Medical Microbiology, Faculty of Medicine, Ege University, İzmir, Turkiye

**Keywords:** Intestinal parasitic infections, solid tumors, diarrhea, parasite detection, cancer treatment

## Abstract

**Background/aim:**

Intestinal parasites are a significant cause of morbidity and mortality in immunosuppressed individuals. This study aimed to evaluate the frequency, distribution, and associated clinical factors of intestinal parasite detection among patients with solid tumors who presented with diarrhea over a 10-year period.

**Materials and methods:**

This retrospective study included patients aged ≥18 years with solid tumors who presented with diarrhea and had stool samples examined at the Ege University Medical Parasitology Laboratory between January 2014 and September 2024. Patients with incomplete records or inadequate stool processing were excluded. Demographic, clinical, and treatment data were retrieved from electronic medical records and analyzed using SPSS version 22.0 (IBM Corp., Armonk, NY, USA).

**Results:**

Among 154 patients (mean age, 57.2 ± 16.3 years; 57.1% female), intestinal parasites were detected in 22.1% of patients (n = 34), with *Cryptosporidium* spp. (16.9%) and *Entamoeba* spp. or the *E. histolytica/E. dispar* complex (2.6%) being the most frequently identified organisms. *Cryptosporidium* spp. showed a borderline, nonsignificant trend toward a higher detection rate among patients with gastrointestinal system (GIS) malignancies (p = 0.067). No clinical or treatment-related variable was significantly associated with overall intestinal parasite detection in either categorical comparisons or univariate logistic regression. However, borderline, nonsignificant trends were observed for GIS malignancy (OR: 1.994; 95% CI: 0.917–4.336; p = 0.079) and recent hospitalization (OR: 0.482; 95% CI: 0.221–1.050; p = 0.066). In patients with available follow-up, clinical improvement was observed after antiparasitic or symptomatic treatment.

**Conclusion:**

Among patients with solid tumors presenting with diarrhea, intestinal parasitic infections should be considered in the differential diagnosis. Early parasitological evaluation and increased oncologist awareness may facilitate timely treatment, symptom control, and prevention of complications.

## 1. Introduction

Intestinal parasitic infections are common worldwide and may cause severe morbidity and mortality, particularly in immunosuppressed individuals. Patients with solid tumors are at increased risk due to both the underlying disease and the immunosuppressive effects of cancer treatments, including radiotherapy and novel targeted therapies [1]. In addition to cancer therapy, comorbidities and poor hygiene conditions play important roles, with higher infection rates observed in developing countries because of inadequate sanitation. Clinically relevant intestinal protozoa reported in oncology patients include *Cryptosporidium* spp., pathogenic *Entamoeba* species, particularly *E. histolytica*, *Cyclospora* spp., and *Blastocystis* spp. These organisms may contribute to gastrointestinal morbidity in immunocompromised hosts, including prolonged or chronic diarrhea, abdominal pain, weight loss, dehydration, malabsorption, and electrolyte disturbances [2]. Intestinal protozoa may disrupt intestinal barrier integrity and trigger immune-mediated tissue injury, potentially interrupting oncologic treatment and increasing the risk of systemic infection [3,4]. Although *Blastocystis* spp. infection may be asymptomatic and its pathogenic role remains debated, *Cryptosporidium* spp. and *E. histolytica* are well-recognized causes of clinically significant gastrointestinal disease in immunocompromised patients. Recent meta-analytic data also indicate that intestinal parasitic infections remain common among cancer patients, with an estimated global prevalence of 28.3% [5,6].

Despite their clinical importance, data on intestinal parasitic infections among patients with solid tumors remain limited. Reported rates vary by geographic region, patient population, immune status, and diagnostic method [7]. Detection rates are higher in endemic and low-resource settings, including parts of Africa and Asia, and meta-analytic indicate a substantially greater burden among immunocompromised populations, particularly people living with HIV [8].

In view of these knowledge gaps, this 10-year retrospective study aimed to assess the frequency of intestinal parasite detection in patients with solid tumors and to evaluate the clinical and treatment-related factors potentially associated with parasite detection.

## 2. Materials and methods

This retrospective study included 154 adult patients (≥18 years) with solid tumors who attended the Department of Medical Oncology at Ege University Faculty of Medicine between January 2014 and September 2024, presented with diarrhea, and underwent stool examination in the Department of Medical Parasitology. Additional stool samples were evaluated in patients with initially negative results and persistent gastrointestinal symptoms, when available. Ethical approval was obtained from the Ege University Faculty of Medicine Ethics Committee (approval no. 24-9.1T/53; 19 September 2024). Patients with incomplete medical records, inadequate stool processing, or age <18 years were excluded.

Demographic data (age, sex, cancer type), treatment modalities (chemotherapy, immunotherapy, targeted therapies, radiotherapy), and recent treatments (within 1 month before stool collection) were recorded. Targeted therapies included antibody–drug conjugates, tyrosine kinase inhibitors, BRAF/MEK inhibitors, CDK4/6 inhibitors, mTOR inhibitors, angiogenesis inhibitors, hormone therapies, and monoclonal antibodies. Clinical data included diarrhea, recent hospitalization, and antibiotic or steroid use within the preceding month. Adverse events were graded using CTCAE v5.0.

For each patient, at least one stool sample and one cellophane tape sample were examined. When clinically indicated or when symptoms persisted despite an initially negative result, additional stool samples were requested and, if available, examined. Stool samples were processed in the Direct Diagnosis Laboratory according to a standardized protocol, including macroscopic examination; saline and iodine wet mounts; concentration using modified formalin–ethyl acetate sedimentation; microscopic examination of concentrated specimens with iodine and Kinyoun acid-fast staining; and Wheatley-modified trichrome staining of diarrheal or suspicious samples. Cellophane tape samples were examined microscopically for *Enterobius vermicularis*. Additional stains, including modified Ziehl–Neelsen and Giemsa, were performed for immunosuppressed patients. Each sample was independently evaluated by two parasitology specialists under light microscopy at ×100, ×400, and ×1000 magnification for *Cryptosporidium* spp., *Entamoeba* spp*., Cyclospora* spp., *Blastocystis* spp., and *E. vermicularis*. Species identification was based on morphology; molecular or antigen-based tests, including PCR and ELISA, were not performed. *Entamoeba histolytica* was identified morphologically only when trophozoites containing ingested erythrocytes were observed, in accordance with the CDC DPD× criteria^1^. In the absence of erythrophagocytosis, *Entamoeba* isolates were reported as *Entamoeba* spp. or the *E. histolytica/E. dispar* complex. Samples that did not meet predefined acceptance criteria, including inappropriate container use, delayed transport, or missing tape samples, were rejected. All procedures were documented in the laboratory information system, and daily records were maintained for internal quality control. Clinical improvement was defined as the resolution of gastrointestinal complaints, particularly diarrhea, during routine outpatient follow-up, without rehospitalization or the need for repeat antiparasitic therapy.

Statistical analyses were performed using SPSS version 22.0 (IBM Corp., Armonk, NY, USA). Continuous variables were expressed as mean ± SD or median (min–max), and categorical variables as frequencies and percentages. Categorical variables were compared using Pearson’s chi-square test or Fisher’s exact test, as appropriate, according to expected cell counts. Univariate binary logistic regression analyses were performed to estimate odds ratios (ORs) and 95% confidence intervals (CIs) for variables potentially associated with intestinal parasite detection. Because of the limited number of parasite-positive cases, a full multivariable logistic regression model was deemed insufficiently reliable. Therefore, the logistic regression analysis was primarily performed using univariate models. As an exploratory sensitivity analysis, a two-variable logistic regression model including gastrointestinal system (GIS) malignancy and recent hospitalization was performed. All statistical tests were two-sided, and a p < 0.05 was considered statistically significant.

## 3. Results

### 3.1. Baseline characteristics of the overall cohort

A total of 154 patients were included in the study. The mean age was 57.2 ± 16.3 years, and 57.1% were female. The overall prevalence of intestinal parasite detection was 22.1% (n = 34). Among the cohort, 34.4% (n = 53) had GIS malignancies, 19.5% (n = 30) had urogenital tract malignancies, and 16.2% (n = 25) had breast cancer, followed by patients with head and neck malignancies (6.5%, n = 10) and lung malignancies (5.2%, n = 8). In the preceding month, 88.9% of patients (n = 137) had received anticancer treatment. Chemotherapy was the most frequently administered treatment modality (74%), followed by targeted therapy (16.8%) and immunotherapy (11%). Concurrent radiotherapy was administered to nine patients (5.8%).

### 3.2. Parasitic species distribution

**Figure 1:**
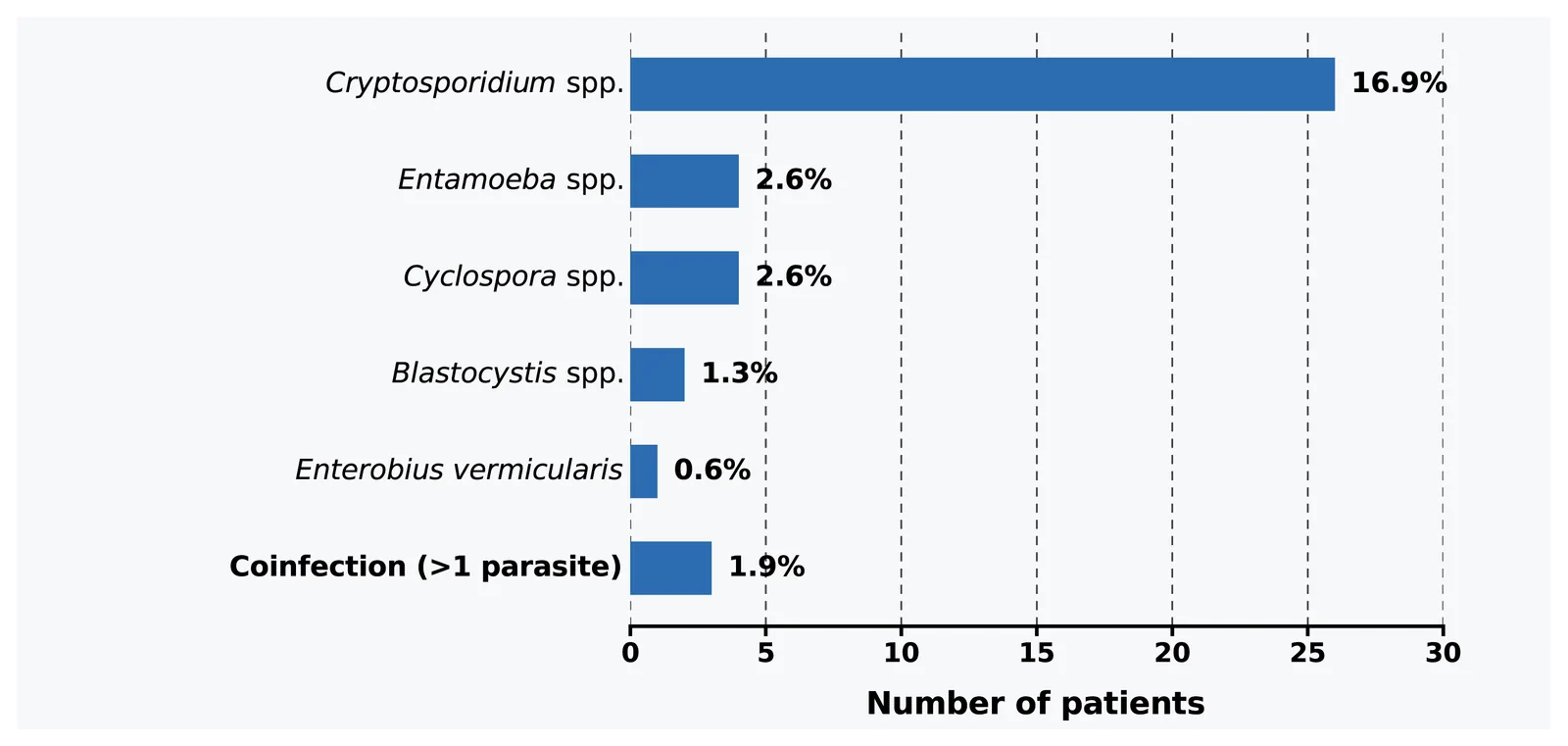
Distribution of detected intestinal parasites among the entire study cohort. Percentages were calculated using the total number of patients who underwent stool examination as the denominator (n = 154). Because coinfections were observed in three patients, parasite-specific categories are not mutually exclusive.

*Cryptosporidium* spp. was the most frequently detected parasite, with a prevalence of 16.9% (n = 26) in the overall study population (n = 154). Other detected parasites included *Entamoeba* spp. or the *E. histolytica/E. dispar* complex (2.6%, n = 4), *Cyclospora* spp. (%2,6; n = 4), *Blastocystis* spp. (1.3%, n = 2), and *Enterobius vermicularis* (0.6%, n = 1). Additionally, three patients (1.9%) had multiple parasite species detected in their stool samples. Figure 1 illustrates the distribution of detected intestinal parasites, with percentages calculated using the overall study cohort (n = 154) as the denominator. Since coinfections were observed in three patients, parasite-specific categories were not mutually exclusive.

### 3.3. Clinical symptoms and associated factors

All included patients were referred for stool examination due to diarrhea. Detailed symptom information was available for 110 patients (71.4%), all of whom had mild to moderate symptoms. In patients with available follow-up, diarrhea resolved with antiparasitic or symptomatic treatment. Among patients presenting with diarrhea, the most commonly administered chemotherapeutic agents were 5-fluorouracil (5-FU), irinotecan, and capecitabine, whereas tyrosine kinase inhibitors were the most commonly administered targeted therapies. Recent antibiotic use was noted in 57.8% of patients (n = 89), and 65.6% (n = 101) had been hospitalized within the past month.

### 3.4. Categorical comparisons and univariate logistic regression analyses

In categorical comparisons using Pearson’s chi-square or Fisher’s exact tests, no clinical or treatment-related variable showed a statistically significant association with overall intestinal parasite detection. GIS malignancy showed a borderline trend toward a higher rate of parasite detection (p = 0.079), whereas recent hospitalization within the last month showed a borderline inverse trend (p = 0.063). The detection rate of *Cryptosporidium* spp. was numerically higher among patients with GIS malignancies than among those with non-GIS malignancies; however, this association did not reach statistical significance (24.5% versus 12.9%; p = 0.067).

**Figure 2:**
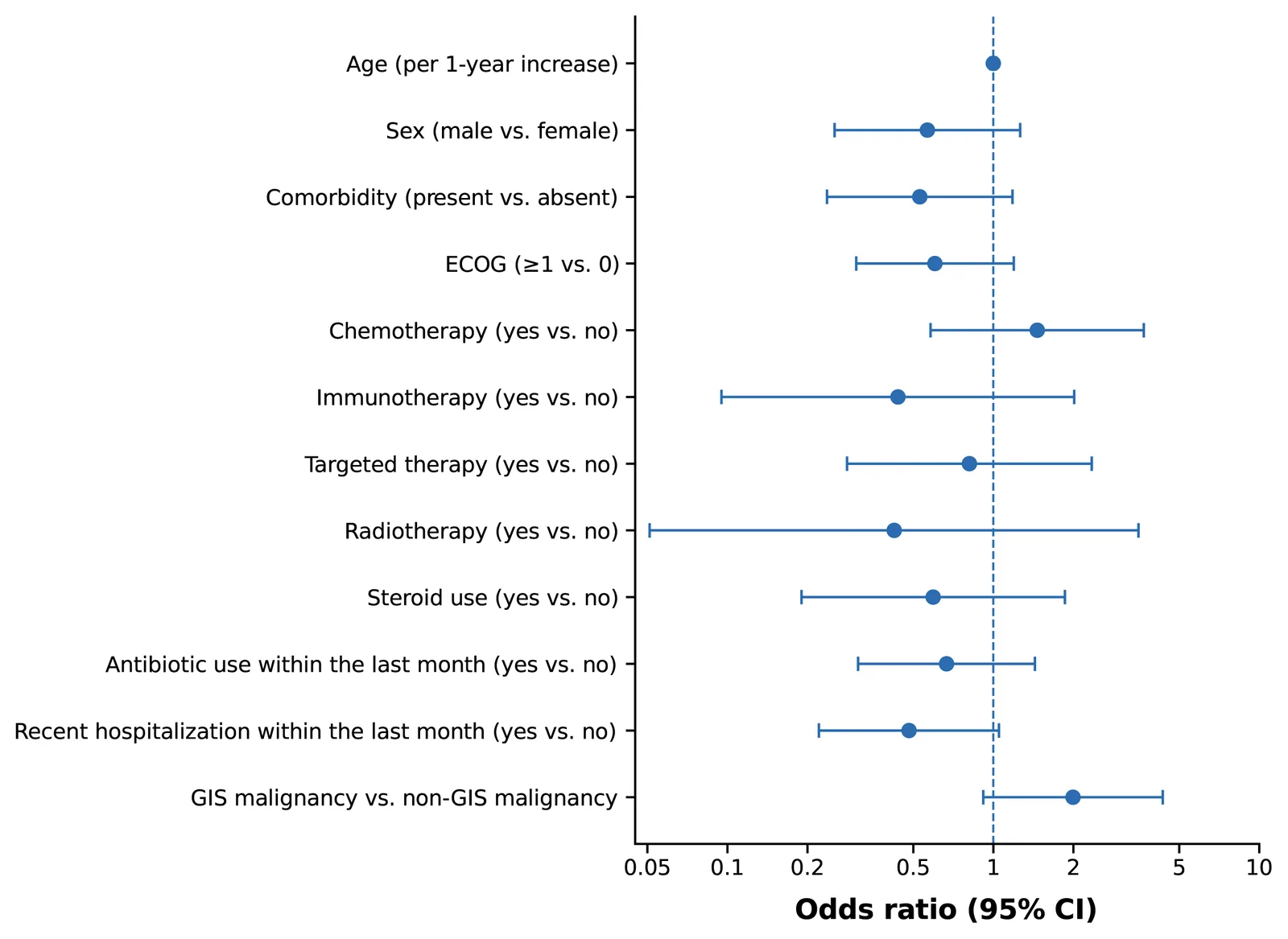
Forest plot of factors evaluated for intestinal parasite detection. Odds ratios (ORs) and 95% confidence intervals (CIs) were obtained from univariate logistic regression analyses. The y-axis lists the clinical and treatment-related variables evaluated in the analysis, and the x-axis shows the odds ratio on a logarithmic scale. Black dots represent OR estimates, and horizontal lines represent the corresponding 95% CIs. The vertical dashed line indicates the null value (OR = 1); confidence intervals that cross this line indicate no statistically significant association. Findings should be interpreted as exploratory because of the limited number of parasite-positive cases.

In univariate logistic regression analyses, none of the evaluated clinical or treatment-related variables showed a statistically significant association with intestinal parasite detection. GIS malignancy showed a borderline trend toward an increased rate of parasite detection (OR: 1.994; 95% CI: 0.917–4.336; p = 0.079), whereas recent hospitalization within the last month showed a borderline inverse association (OR: 0.482; 95% CI: 0.221–1.050; p = 0.066). The results of the categorical comparisons and univariate logistic regression analyses are summarized in Tables 1 and 2, respectively, and a forest plot of odds ratios for evaluated variables is shown in Figure 2. In an exploratory two-variable logistic regression model including GIS malignancy and recent hospitalization, neither variable remained statistically significant. Recent hospitalization showed a borderline inverse association (adjusted OR: 0.496; 95% CI: 0.226–1.089; p = 0.081), whereas GIS malignancy showed a nonsignificant trend toward an increased rate of parasite detection (adjusted OR: 1.935; 95% CI: 0.882–4.246; p = 0.100).

### 3.5. Treatment of parasitic infections

Among patients with detected Cryptosporidium spp., 65.4% (n = 17) received azithromycin, and 7.7% (n = 2) received metronidazole. Antiparasitic treatment was not administered to seven patients because they did not attend follow-up visits after the initial assessment. Among patients with detected *Entamoeba* spp. or the *E. histolytica/E. dispar* complex, three patients were treated with metronidazole, whereas one patient remained untreated for the same reason. As all included patients presented with diarrhea, untreated patients were not asymptomatic at baseline; however, their subsequent clinical course, including spontaneous symptom resolution or persistence of diarrhea, could not be evaluated because follow-up data were unavailable.

## 4. Discussion

Intestinal parasites are an important cause of diarrhea in both immunocompetent and immunosuppressed individuals, with cancer patients being particularly vulnerable because of the underlying disease, cancer treatments, comorbidities, and hygiene-related factors. In our cohort, intestinal parasites were detected in 22.1% of patients. Previous studies in cancer populations have reported variable prevalence rates, ranging from 8% to 38.7%, depending on geographic region, cancer type, treatment status, hygiene-related factors, and diagnostic methods [2,5,9]. In a Turkish study of patients with gastric cancer, intestinal parasite detection rates were significantly higher than those in healthy controls (14% versus 2%, respectively); *Blastocystis* spp. was the most frequently detected parasite, followed by *Cryptosporidium*, *Giardia intestinalis*, and *Cyclospora cayetanensis* [10]. A recent Malaysian study also reported intestinal parasitic infections in 32.8% of cancer patients and demonstrated significant gut microbiota differences between patients with and without parasitic infections [2].

In our cohort, *Cryptosporidium* spp. was the most frequently identified parasite, whereas *Blastocystis* spp., commonly reported as predominant in previous studies, was detected at a low frequency. In line with our findings, a recent study from Nepal also reported *Cryptosporidium* as the most frequently detected parasite among patients with cancer, although the overall prevalence was lower than that observed in our cohort [9]. *Entamoeba* spp. or the *E. histolytica/E. dispar* complex was detected in only 2.6% of patients. These differences may reflect regional epidemiology, patient characteristics, prior antimicrobial exposure, or variations in diagnostic approaches. The low detection rate of *Blastocystis* spp. may partly reflect the limited sensitivity of microscopy, as recent studies have reported markedly higher detection rates of *Blastocystis* and *Cryptosporidium* using PCR-based methods [11]. Because PCR-based testing was not performed in our study, underdetection of *Blastocystis* spp. and other protozoa cannot be excluded.

GIS malignancy showed a borderline, nonsignificant trend toward higher intestinal parasite detection in both categorical comparisons and univariate logistic regression analysis (OR: 1.994; 95% CI: 0.917–4.336; p = 0.079). Similarly, in the exploratory two-variable logistic regression model, GIS malignancy remained nonsignificant (adjusted OR: 1.935; 95% CI: 0.882–4.246; p = 0.100). Therefore, GIS malignancy should not be interpreted as an established risk factor; this finding should be considered exploratory and requires confirmation in larger cohorts. Nevertheless, the gastrointestinal tract is the primary site of entry and colonization by intestinal parasites, and mucosal disruption, altered microbiota, or treatment-related injury may increase susceptibility in selected patients [12].

In the present study, *Cryptosporidium* spp. was the most frequently detected parasite and showed a borderline, nonsignificant trend toward higher detection rates among patients with GIS malignancies than among those with non-GIS malignancies (24.5% versus 12.9%; p = 0.067). This apicomplexan protozoan is well documented as a cause of severe gastrointestinal disease in immunocompromised hosts, including patients with cancer [13]. The combination of gastrointestinal tract vulnerability and treatment-related immunosuppression may contribute to this finding. Similar patterns have been reported in other oncology cohorts, where *Cryptosporidium* spp., *Giardia lamblia*, and *Cystoisospora belli* were identified as notable pathogens [14]. Given its potential to complicate cancer management, particularly in symptomatic or immunosuppressed patients, targeted stool examination may be considered in selected high-risk patients.

None of the evaluated treatment-related variables, including chemotherapy, immunotherapy, and targeted therapy, showed a statistically significant association with overall intestinal parasite detection in our cohort. Chemotherapy was not significantly associated with parasite detection in univariate logistic regression analysis (OR: 1.463; 95% CI: 0.581–3.682; p = 0.419), although chemotherapy-related mucosal barrier injury, microbiota disruption, and immunosuppression may increase susceptibility to enteric infections in selected patients. Previous studies have reported the detection of *Cryptosporidium* spp. in chemotherapy-treated patients with solid tumors and diarrhea, supporting consideration of this pathogen in symptomatic patients [2,15]. Similarly, no significant association was observed with immunotherapy; however, diarrhea in immune checkpoint inhibitor (ICI)-treated patients may result from either immune-mediated colitis or infectious etiologies. In a recent study of 521 ICI-treated patients with diarrhea, stool PCR identified enteric pathogens in 61 patients, including *Giardia lamblia*, *Cryptosporidium* spp., and *Entamoeba histolytica* [16]. Targeted therapy was also not significantly associated with parasite detection (OR: 0.813; 95% CI: 0.282–2.345; p = 0.701). Given the limited number of parasite-positive cases and the heterogeneity of treatment exposures, these findings should be interpreted cautiously.

Although *Cryptosporidium* spp. can cause severe or prolonged diarrhea in immunocompromised patients, no severe cases were identified within this cohort. Several anticancer treatments, such as 5-FU, irinotecan, and tyrosine kinase inhibitors, are also associated with gastrointestinal toxicity, which complicates the differentiation between parasitic infection and treatment-related diarrhea. Parasitic infections should be considered in patients with cancer who present with diarrhea that is persistent, atypical, or inconsistent with the timing of treatment, as these infections may coexist with or mimic treatment-related gastrointestinal toxicity [17,18]. No significant association was observed between antibiotic or steroid use and parasite detection. Recent hospitalization demonstrated a borderline inverse association with parasite detection in both categorical comparisons and univariate logistic regression; however, neither reached statistical significance (p = 0.063 and p = 0.066, respectively). These findings warrant cautious interpretation, as they may reflect variable exposure patterns, residual confounding, empiric antimicrobial exposure, or the limited number of parasite-positive cases. More broadly, gastrointestinal infections may contribute to adverse outcomes, including mortality, among hospitalized patients [19].

Azithromycin was administered to 65.4% of patients with *Cryptosporidium* spp. detection, and clinical improvement was observed in patients with available follow-up [6]. Although nitazoxanide is an FDA-approved treatment option for cryptosporidiosis, its efficacy may be limited in immunocompromised patients, and its limited availability in Türkiye may have influenced treatment preferences in our cohort [20]. Patients with *Entamoeba* spp. or the *E. histolytica/E. dispar* complex were treated with metronidazole in accordance with routine clinical practice, based on gastrointestinal symptoms, immunosuppression, and clinical judgment, despite the absence of definitive molecular differentiation [21]. However, because nonpathogenic *Entamoeba* species cannot be reliably excluded by microscopy alone, treatment decisions and clinical outcomes in such cases should be interpreted with caution.

## 5. Limitations and strengths

The main limitation of this study is its retrospective, single-center design, which may reduce the generalizability of the findings and introduce selection bias. Long-term follow-up data were not consistently available, preventing a reliable assessment of recurrence or reinfection. Another important limitation is the relatively small sample size despite the 10-year study period. Since this was a laboratory-based retrospective study, only patients whose stool samples were referred to the Department of Medical Parasitology and met the laboratory acceptance criteria were included. Therefore, the cohort represents a selected subgroup of patients with solid tumors and diarrhea, rather than all patients with diarrhea treated at our tertiary center. This may have reduced the precision of the prevalence estimates and limited the statistical power of the categorical comparisons and logistic regression analyses, particularly for rare associations between parasite detection and treatment exposure.

Stool testing was performed based on symptoms, which may have led to underestimation of the true prevalence by missing asymptomatic carriers. Because only patients who underwent stool examination for diarrhea were included, parasite detection rates could not be compared with those in asymptomatic solid tumor patients or in patients without gastrointestinal complaints. Therefore, the causal contribution of intestinal parasites to diarrhea cannot be definitively established. Another limitation is the absence of molecular, serological, or antigen-based diagnostic methods, such as PCR or ELISA, which may have limited species-level identification, particularly for *Entamoeba* and *Cryptosporidium*. In the absence of erythrophagocytosis, microscopy alone cannot reliably distinguish pathogenic *E. histolytica* from morphologically similar nonpathogenic species such as *E. dispar*; therefore, these isolates should be interpreted as the *E. histolytica/E. dispar* complex rather than confirmed *E. histolytica* infection. Similarly, the lack of PCR or antigen-based confirmation for *Cryptosporidium* prevented species-level identification, potentially limiting the epidemiological interpretation of the findings and the interpretation of treatment-related outcomes. Because of the limited number of parasite-positive cases, a full multivariable model was not considered reliable; therefore, the regression analyses were primarily performed using univariate models, and the exploratory two-variable model should be interpreted as hypothesis-generating.

Despite these limitations, this study has several strengths. It evaluated a clinically relevant cohort of patients with solid tumors at a tertiary care center over a 10-year period, allowing for relatively consistent clinical and laboratory assessment. Parasitological examinations were performed by experienced specialists using standardized workflows, supporting the internal validity of the findings. In addition, the inclusion of clinical variables such as recent treatments, hospitalization status, antibiotic and steroid exposure, and symptom profiles allowed the exploration of factors that may be relevant to daily oncology practice. These findings provide useful epidemiological insights and support the need for larger, prospective, multicenter studies that incorporate molecular diagnostics and more comprehensive clinical follow-up.

## 6. Conclusion

In conclusion, intestinal parasitic infections should be considered in patients with solid tumors presenting with diarrhea. Timely stool examination, referral for parasitological evaluation, and increased oncologist awareness may help improve early diagnosis and treatment in symptomatic patients. Although GIS malignancy and recent hospitalization showed borderline, nonsignificant trends, these findings require confirmation in larger cohorts. Prospective multicenter studies incorporating molecular diagnostics are needed to clarify the true prevalence, species distribution, and clinical impact of intestinal parasites in this population.

## Figures and Tables

**Table 1:** Association between intestinal parasite detection and clinical variables in patients with solid tumors.

Clinical variable	p-value
GIS malignancy vs non-GIS malignancy	0.079
Sex	0.161
Diabetes mellitus	0.372
Hypertension	0.892
Chemotherapy	0.417
Targeted therapy	0.701
Immunotherapy	0.365*
Radiotherapy	0.685*
Antibiotic use within the last month	0.297
Steroid use	0.367
Recent hospitalization within the last month	0.063

Footnote: Categorical variables were compared using Pearson’s chi-square test or Fisher’s exact test, as appropriate, according to expected cell counts. Fisher’s exact test was used when expected cell counts were <5. A p < 0.05 was considered statistically significant.

* Fisher’s exact test.

Abbreviations: GIS, gastrointestinal system; p, probability value.

**Table 2:** Univariate logistic regression analysis of clinical and treatment-related factors associated with intestinal parasite detection in patients with solid tumors.

Variable	OR (95% CI)	p-value
Age, per 1-year increase	1.000 (0.977–1.024)	0.992
Sex (male vs. female)	0.565 (0.253–1.262)	0.164
Comorbidity (present vs. absent)	0.529 (0.237–1.180)	0.120
ECOG (≥1 vs. 0)	0.603 (0.305–1.194)	0.147
Chemotherapy (yes vs. no)	1.463 (0.581–3.682)	0.419
Immunotherapy (yes vs. no)	0.438 (0.095–2.016)	0.289
Targeted therapy (yes vs. no)	0.813 (0.282–2.345)	0.701
Radiotherapy (yes vs. no)	0.424 (0.051–3.516)	0.427
Steroid use (yes vs. no)	0.594 (0.190–1.859)	0.371
Antibiotic use within the last month (yes vs. no)	0.667 (0.310–1.433)	0.299
Recent hospitalization within the last month (yes vs. no)	0.482 (0.221–1.050)	0.066
GIS malignancy vs. non-GIS malignancy	1.994 (0.917–4.336)	0.079

Footnote: ORs and 95% confidence intervals were obtained from univariate binary logistic regression analyses. A p < 0.05 was considered statistically significant. Abbreviations: OR, odds ratio; CI, confidence interval; GIS, gastrointestinal system; ECOG, Eastern Cooperative Oncology Group; p, probability value.

## Data Availability

The data that support the findings of this study are available from the corresponding author upon reasonable request.

## Footnotes

1 Centers for Disease Control and Prevention (2009). Laboratory diagnosis of amebiasis: *Entamoeba histolytica* and *Entamoeba dispar*. DPDx: Laboratory Identification of Parasites of Public Health Concern [online]. Website https://www.cdc.gov/dpdx/resources/pdf/benchaids/entamoeba_benchaid.pdf [accessed 15 June 2026].
